# The treatment outcomes of antiretroviral substitutions in routine clinical settings in Asia; data from the TREAT Asia HIV Observational Database (TAHOD)

**DOI:** 10.1002/jia2.25016

**Published:** 2017-12-15

**Authors:** In Young Jung, David Boettiger, Wing Wai Wong, Man Po Lee, Sasisopin Kiertiburanakul, Romanee Chaiwarith, Anchalee Avihingsanon, Junko Tanuma, Nagalingeswaran Kumarasamy, Adeeba Kamarulzaman, Fujie Zhang, Pacharee Kantipong, Oon Tek Ng, Benedict Lim Heng Sim, Matthew Law, Jeremy Ross, Jun Yong Choi

**Affiliations:** ^1^ Department of Internal Medicine Yonsei University College of Medicine Seoul South Korea; ^2^ AIDS Research Institute Yonsei University College of Medicine Seoul South Korea; ^3^ The Kirby Institute UNSW Sydney Sydney Australia; ^4^ Taipei Veterans General Hospital Taipei Taiwan; ^5^ Queen Elizabeth Hospital Hong Kong China; ^6^ Faculty of Medicine Ramathibodi Hospital Mahidol University Bangkok Thailand; ^7^ Research Institute for Health Sciences Chiang Mai Thailand; ^8^ HIV‐NAT/Thai Red Cross AIDS Research Centre Bangkok Thailand; ^9^ National Center for Global Health and Medicine Tokyo Japan; ^10^ Chennai Antiviral Research and Treatment Clinical Research Site (CART CRS) YRGCARE Medical Centre VHS Chennai India; ^11^ University Malaya Medical Centre Kuala Lumpur Malaysia; ^12^ Beijing Ditan Hospital Capital Medical University Beijing China; ^13^ Chiangrai Prachanukroh Hospital Chiang Rai Thailand; ^14^ Tan Tock Seng Hospital Singapore Singapore; ^15^ Hospital Sungai Buloh Sungai Buloh Malaysia; ^16^ TREAT Asia, amfAR – The Foundation for AIDS Research Bangkok Thailand

**Keywords:** ART, substitution, Asian countries, clinical failure, virological failure, effectiveness

## Abstract

**Introduction:**

Although substitutions of antiretroviral regimen are generally safe, most data on substitutions are based on results from clinical trials. The objective of this study was to evaluate the treatment outcomes of substituting antiretroviral regimen in virologically suppressed HIV‐infected patients in non‐clinical trial settings in Asian countries.

**Methods:**

The study population consisted of HIV‐infected patients enrolled in the TREAT Asia HIV Observational Database (TAHOD). Individuals were included in this analysis if they started combination antiretroviral treatment (cART) after 2002, were being treated at a centre that documented a median rate of viral load monitoring ≥0.8 tests/patient/year among TAHOD enrolees, and experienced a minor or major treatment substitution while on virally suppressive cART. The primary endpoint to evaluate outcomes was clinical or virological failure (VF), followed by an ART class change. Clinical failure was defined as death or an AIDS diagnosis. VF was defined as confirmed viral load measurements ≥400 copies/mL followed by an ART class change within six months. Minor regimen substitutions were defined as within‐class changes and major regimen substitutions were defined as changes to a drug class. The patterns of substitutions and rate of clinical or VF after substitutions were analyzed.

**Results:**

Of 3994 adults who started ART after 2002, 3119 (78.1%) had at least one period of virological suppression. Among these, 1170 (37.5%) underwent a minor regimen substitution, and 296 (9.5%) underwent a major regimen substitution during suppression. The rates of clinical or VF were 1.48/100 person years (95% CI 1.14 to 1.91) in the minor substitution group, 2.85/100 person years (95% CI 1.88 to 4.33) in the major substitution group and 2.53/100 person years (95% CI 2.20 to 2.92) among patients that did not undergo a treatment substitution.

**Conclusions:**

The rate of clinical or VF was low in both major and minor substitution groups, showing that regimen substitution is generally effective in non‐clinical trial settings in Asian countries.

## 1 Introduction

Combination antiretroviral treatments (cART) have been widely available in Asia since 2003 1. However, most Asian HIV clinics have limited resources and are able to prescribe regimens based on WHO global treatment guidelines, which recommend dual nucleoside reverse transcriptase inhibitors (NRTIs) plus a non‐nucleoside reverse transcriptase inhibitor (NNRTI) for first‐line therapy 1. Current WHO guidelines recommend use of a ritonavir‐boosted protease inhibitor (PI) in combination with dual NRTIs after failure on a first line NNRTI‐based regimen, which is widely practiced in Asia 2. A prior TREAT Asia HIV Observational Database (TAHOD) study reported that among 302 patients with first‐line treatment failure, 73% switched to a dual NRTI plus boosted PI regimen 3. Use of ritonavir‐boosted lopinavir (LPV/r) or atazanavir (ATV/r) compromised the majority of boosted PI use beyond 2006 in this cohort 3. Most commonly used NRTIs for second‐line treatment were lamivudine/emtricitabine (3TC/FTC), tenofovir (TDF) and zidovudine (AZT), accounting for 76.5%, 44.4% and 32.1% of all patients respectively 3.

Prior to 2003, PI‐based ART was more commonly used in small numbers of patients 1. The availability of generic nevirapine (NVP) and efavirenz (EFV) allowed expansion of cART due to supply and cost 4. In a cohort study of 4662 patients treated in Asia, stavudine (d4T) plus another NRTI plus NNRTI was the most common first‐line regimen used from 2003 to 2006 1. However, between 2003 and 2013 first line d4T use decreased from 68.2% to 5.8% in the cohort, frequently because of side effects such as lipodystrophy and peripheral neuropathy 1. Currently, the region is phasing out d4T use according to the WHO 2010 recommendations 5. More recently, integrase inhibitors (INSTIs) including dolutegravir (DTG) and elvitegravir (EVG) have begun to be introduced 6.

Despite great advances in antiretroviral therapy in the last decade, several limitations remain including adverse effects, suboptimal adherence, tolerability problems and drug–drug interactions 7, 8, 9. Substitution of cART in stable, virologically suppressed patients with the aim of improving tolerability and convenience is a common practice in clinical settings 10. Data on the safety and durability of virological suppression following switches within or across antiretroviral classes have largely come from randomized controlled trials. For example the SPIRAL study demonstrated non‐inferior efficacy in switching from ritonavir‐boosted PI to raltegravir (RAL) 11, the EASIER ANRS 138 trial demonstrated that switch from enfuvirtide to RAL in well‐suppressed patients with multidrug‐resistant HIV infection was generally well tolerated and had sustained efficacy 12, and the STRATEGY‐NNRTI trial demonstrated non‐inferiority in switching to co‐formulated elvitegravir‐cobicistat‐emtricitabine‐tenofovir (ECF/TDF) versus continuation NNRTI with FTC and TDF 13.

However, data on the outcomes of these switches from real‐world clinic data have been lacking. Therefore, the objective of this study was to evaluate the treatment outcomes of substituting antiretroviral regimen in virologically suppressed HIV‐infected patients in non‐clinical trial settings in Asian countries.

## 2 Methods

### 2.1 Patient selection

The study population consisted of HIV‐infected patients enrolled in the TAHOD before March 30, 2016. This cohort contributes to the International Epidemiology Databases to Evaluate AIDS (IeDEA) global consortium and has been described previously 14, 15, 16. Recruitment started in 2003. In March 2016, TAHOD included data from 8928 adults (≥18 years of age) that had ever received care from one of 20 clinics in 12 Asian countries. These sites are predominantly public or university‐based HIV referral clinics. Ethics approval is obtained at the sites, TREAT Asia/amfAR (coordinating centre), and the Kirby Institute (data management and statistical analysis centre). Patient consent was deferred according to the individual participating sites and their institutional review boards, and is not required for all participants. Individuals were included in this analysis if they started cART after 2002, were being treated at a centre that documented a median rate of viral load monitoring ≥0.8 tests/patient/year among TAHOD enrolees, and experienced a minor or major treatment switch while on virally suppressive ART. Among the 20 cohort sites, only data from the 12 sites which performed VL test rate >0.8 in every year were included in this analysis: Hong Kong (n = 1), India (n = 1), Japan (n = 1), Malaysia (n = 2), Singapore (n = 1), South Korea (n = 1), Taiwan (n = 1) and Thailand (n = 4).

### 2.2 Baseline data and outcome definitions

Two analyses were conducted to evaluate minor and major treatment substitutions separately. Viral suppression was defined as having had two viral load measurements <400 copies/mL between 90 to 390 days apart. Periods of viral suppression were considered to begin from the date of the second viral load <400 copies/mL. Minor regimen substitutions were defined as within‐class changes (e.g. d4T to TDF, EFV to NVP) and major regimen substitutions were defined as changes to a drug class (e.g. NNRTI to PI, PI to INSTI). Baseline time in both analyses (minor switches and major switches) was defined as the date of first ART substitution after achieving viral suppression. Patients who had never received mono/dual therapy prior to baseline were excluded. For an approximate reference point, we also evaluated rates of VF among patients who never underwent an ART switch. The baseline date used to calculate follow‐up time for the non‐switch group was the date of viral suppression. The primary endpoint to evaluate outcomes was clinical or virological failure (VF), followed by an ART class change. Clinical failure was defined as death or an AIDS diagnosis. VF was defined as confirmed viral load measurements ≥400 copies/mL followed by an ART class change within six months 17, 18.

The reason for treatment substitution was categorized as adverse event (AE)‐associated when a patient had any documentation of an adverse event‐associated treatment substitution (as determined by the treating physician) between the date of viral suppression and baseline. Patients were considered hepatitis B (HBV) co‐infected if they had any record of a positive HBV surface antigen test, and hepatitis C (HCV) co‐infected if they had any record of a positive HCV antibody test. The window period for baseline CD4 cell count testing was between three months before baseline to three months after baseline. Where multiple test results were available within this period, the measurement closest to the time of treatment substitution was used. Nadir CD4 cell count was defined as the lowest documented CD4 cell count prior to baseline. Peak HIV‐1 RNA level (viral load, VL) was defined as the highest documented VL prior to baseline. Country income status was defined according to World Bank categorizations 19. Loss to follow‐up was defined as not having been seen at clinic for >6 months without documentation of clinic transfer.

### 2.3 Statistical analysis

The rates of virological failure after treatment substitutions were calculated. Competing risk regression was used to determine factors associated with VF after treatment substitution. Loss to follow up and major treatment substitution after baseline were considered competing risks. Type of treatment substitution, reason for treatment substitution (adverse event or other), baseline age, sex, HIV exposure category, HBV surface antigen/HCV antibody positivity, AIDS diagnosis prior to baseline, nadir CD4 count, peak viral load, period of treatment substitution and country income status were evaluated as fixed covariates. Co‐trimoxazole use, CD4 cell count and ART adherence were evaluated as time‐updated covariates. The final models included type of ART substitution, baseline age, sex, CD4 cell count, ART adherence, period of treatment substitution and country income status. Any other variables found to be significant after adjusting for these core variables were also included. Patients with missing data were included in all analyses but hazard ratios for missing categories are not reported. Stata (StataCorp, College Station, TX) version 14.1 was used for all statistical analysis.

## 3 Results

Of the 3994 adults who started ART after 2002, 3119 (78.1%) had at least one period of virological suppression. Among these, 296 (9.5%) underwent a major regimen substitution during suppression, and 1170 (37.5%) underwent a minor regimen substitution during suppression. A total of 17 patients in the minor switch, six in the major switch and 69 in the non‐switch group were confirmed with two consecutive viral load measurements ≥400 copies/mL. Patients confirmed with a single viral load ≥400 copies/mL were 13, 3 and 28 in the minor, major and non‐switch group respectively. AIDS diagnosis was confirmed in 14, 7 and 65 patients in the minor, major and non‐switch group respectively. Death was confirmed in 15, 6 and 31 patients in the minor, major and non‐switch group respectively.

The baseline characteristics of the study participants are described in Table 1. In the minor and major switch groups, the median (IQR) age of the patients were 41.8 (36 to 48), and 42.7 (35.8 to 50.3) years respectively. Seven hundred and eighty‐two (66.8%) were male in the minor switch group, and 250 (84.5%) in the major switch group. Heterosexual transmission accounted for 788 (67.4%), and 109 (36.8%) in the minor and major switch groups. Homosexual transmission accounted for 294 (25.1%), and 157 (53.0%) in each groups. The CD4 cell count was 432 (300 to 589) cells/mm^3^ in the minor switch group, and 490 cells/mm^3^ (338 to 643) in the major switch group. 618 (52.8%) and 158 (53.4%) switches were associated with an adverse events in the minor and major switch groups respectively.

**Table 1:** Baseline characteristics at minor and major regimen switch

Characteristic	Minor switch group N = 1170		Major switch group N = 296	
Age (years)				
Median (IQR)	41.8	(36.0 to 48.0)	42.7	(35.8to 50.3)
Sex				
Male	782	66.8%	250	84.5%
HIV exposure				
Heterosexual	788	67.4%	109	36.8%
Homosexual	294	25.1%	157	53.0%
IDU	20	1.7%	2	0.7%
Other	68	5.8%	28	9.5%
Hepatitis B surface antigen status				
Negative, % tested	979	90.1%	243	91.7%
Positive, % tested	108	9.9%	22	8.3%
Unknown	83	7.1%	31	10.5%
Hepatitis C antibody status				
Negative, % tested	961	93.2%	255	95.5%
Positive, % tested	70	6.8%	12	4.5%
Unknown	139	11.9%	29	9.8%
Prior AIDS diagnosis				
Yes	525	44.9%	102	34.5%
Using cotrimoxazole prophylaxis				
Yes	114	9.7%	19	6.4%
CD4 cell count (cells/mm^3^)				
Median (IQR)	432	(300 to 589)	490	(338 to 643)
Number tested	1019	87.1%	271	91.6%
Nadir CD4 cell count (cells/mm^3^)				
Median (IQR)	107	(33 to 209)	170	(77 to 271)
Number tested	1170	100.0%	295	99.7%
Peak HIV viral load (copies/mL)				
Median (IQR)	101,500	(37,800 to 365,000)	100,501	(32,225 to 313,500)
Number tested	822	70.3%	236	79.7%
Time on ART (years)				
Median (IQR)	3.2	(1.8 to 4.9)	3.5	(2.1 to 6.9)
Duration of suppression (years)				
Median (IQR)	1.8	(0.7 to 3.4)	2.8	(1.3 to 6.2)
Adverse event‐associated switch				
Yes	618	52.8%	158	53.4%
Period of treatment switch				
2003 to 2006	142	12.1%	13	4.4%
2007 to 2009	238	20.3%	41	13.9%
2010 to 2012	482	41.2%	105	35.5%
2013 to 2016	308	26.3%	137	46.3%
Country income status				
High	374	32.4%	218	74.4%
Middle/low	780	67.6%	75	25.6%

Values are n (% total) unless otherwise indicated. ART, antiretroviral therapy.

Table 2 describes the switch characteristics in each group. In the minor substitution group, 825 patients switched between NRTI only, 70 patients switched between NNRTI only, 101 patients switched between PI only, three patients switched between INSTI only and 171 patients experienced multiple within class substitutions. In the major substitution group, 78 patients switched from NNRTI to PI, 88 patients switched from PI to NNRTI, 95 patients switched from PI to INSTI and 35 patients experienced other regimen switch. The number of patients in the minor switch group who switched due to adverse events was 446 in the NRTI only group, 39 in the NNRTI only group, 50 in the PI only group, 1 in the INSTI only group and 82 in patients who had multiple within class switches. The number of patients who experienced major switch due to adverse events was 45 in the NNRTI to PI group, 36 in the PI to NNRTI group, 64 in the PI to INSTI group and 13 in the other regimen substitution group.

**Table 2:** Minor and Major regimen switch characteristics

Switch class description	Number of switches	Median (IQR) CD4 cell count at switch (cells/mm^3^), number with measurement	Median (IQR) duration of HIV suppression at switch (years)	Number of switches associated with AE (% within switch category)
Minor				
NRTI only	825	436 (303 to 593), n = 709	1.8 (0.7 to 3.5)	446 (54.1)
NNRTI only	70	430 (272 to 605), n = 63	1.8 (0.7 to 3.5)	39 (55.7)
PI only	101	443 (291 to 579), n = 89	1.8 (0.6 to 2.9)	50 (49.5)
INSTI only	3	485 (474 to 504), n = 3	0.5 (0.3 to 2.8)	1 (33.3)
Multiple within class switches	171	388 (292 to 579), n = 155	1.8 (0.7 to 3.6)	82 (48.0)
Major				
NRTI only	78	397 (249 to 547), n = 71	1.7 (0.7 to 3.5)	45 (57.7)
NNRTI only	88	514 (382 to 632), n = 80	3.2 (1.5 to 6.9)	36 (40.9)
PI only	95	580 (426 to 687), n = 89	4.3 (2.0 to 7.2)	64 (67.4)
Other	35	365 (272 to 574), n = 31	2.2 (1.2 to 5.2)	13 (37.1)

NRTI, nucleoside reverse transcriptase inhibitors; NNRTI, non‐nucleoside reverse transcriptase inhibitor; PI, protease inhibitor; INSTI, integrase inhibitors; AE, adverse events.

The rates of VF were 2.85 per 100 person years (95% CI 1.88 to 4.33) in the major substitution group and 1.48 per 100 person years (95% CI 1.14 to 1.91) in the minor substitution group. The rate of VF among patients that did not undergo any treatment substitution during suppression (n = 1756) was 2.53 per 100 person years (95% CI 2.20 to 2.92). The median (IQR) duration of follow‐up was 3.1 (1.3 to 5.1) years in the minor switch analysis and 1.8 (0.9 to 3.7) years in the major switch analysis. Rates of loss to follow up were 1.7 per 100 patient years (95% CI 1.3 to 2.1) and 1.7 per 100 patient years (95% CI 1.0 to 2.9) respectively. Tables 3 and 4 demonstrate the crude risk and hazard ratios for VF with different patterns of minor and major switch. The results in both groups were similar, showing both groups of switching were not a significant risk factor for VF.

**Table 3:** Crude risk and hazard ratios for virological failure with different patterns of minor ART switch

Covariate	VF	Patient years follow up	Rate per 100 patient/years (95% CI)	Univariate HR (95% CI)	*p*	Multivariate HR (95% CI)	*p*
Type of minor ART switch^a^							
NRTI only	43	2885.8	1.49 (1.11 to 2.01)	1.00		1.00	
NNRTI only	2	176.5	1.13 (0.28 to 4.53)	0.65 (0.16 to 2.67)	0.55	0.55 (0.13 to 2.35)	0.42
PI only	7	376.5	1.86 (0.89 to 3.90)	1.38 (0.62 to 3.03)	0.43	1.40 (0.57 to 3.42)	0.46
INSTI only or multiple within class switches	7	558.3	1.25 (0.60 to 2.63)	0.78 (0.35 to 1.74)	0.55	0.63 (0.28 to 1.40)	0.26
AE associated switch							
No	30	1877.0	1.60 (1.12 to 2.29)	1.00			
Yes	29	2120.2	1.37 (0.95 to 1.97)	0.87 (0.52 to 1.44)	0.59		
Baseline age^a^							
Per 5 years older	59	3997.2	1.48 (1.14 to 1.91)	1.10 (0.98 to 1.23)	0.12	1.09 (0.96 to 1.23)	0.19
Sex^a^							
Male	47	2670.0	1.76 (1.32 to 2.34)	1.00		1.00	
Female	12	1327.2	0.90 (0.51 to 1.59)	0.50 (0.26 to 0.95)	0.03	0.66 (0.35 to 1.27)	0.21
HIV exposure							
Heterosexual	37	2641.8	1.40 (1.01 to 1.93)	1.00			
Homosexual	16	1052.9	1.52 (0.93 to 2.48)	1.20 (0.67 to 2.14)	0.55		
IDU/other	6	302.5	1.98 (0.89 to 4.42)	1.38 (0.58 to 3.28)	0.46		
Hepatitis B surface antigen status							
Negative	52	3304.1	1.57 (1.20 to 2.07)	1.00			
Positive	5	402.8	1.24 (0.52 to 2.98)	0.84 (0.33 to 2.12)	0.71		
Unknown	2	290.3	0.69 (0.17 to 2.75)	–			
Hepatitis C antibody status							
Negative	50	3312.9	1.51 (1.14 to 1.99)	1.00			
Positive	6	211.6	2.84 (1.27 to 6.31)	1.64 (0.71 to 3.83)	0.25		
Unknown	3	472.8	0.63 (0.20 to 1.97)	–			
Prior AIDS diagnosis							
No	31	2145.7	1.44 (1.02 to 2.05)	1.00			
Yes	28	1851.5	1.51 (1.04 to 2.19)	1.09 (0.65 to 1.81)	0.74		
Current cotrimoxazole use							
No	56	3765.5	1.49 (1.14 to 1.93)	1.00			
Yes	3	231.7	1.29 (0.42 to 4.01)	0.73 (0.23 to 2.33)	0.59		
Current CD4 cell count (cells/mm^3^)^a^							
>500	17	1832.3	0.93 (0.58 to 1.49)	1.00		1.00	
350 to 500	17	1122.3	1.51 (0.94 to 2.44)	1.48 (0.75 to 2.92)	0.25	1.39 (0.68 to 2.83)	0.37
<350	21	926.7	2.27 (1.48 to 3.48)	1.93 (1.01 to 3.70)	0.05	1.59 (0.79 to 3.21)	0.19
Unknown	4	115.8	3.45 (1.30 to 9.20)	–		–	
Nadir CD4 cell count (cells/mm^3^)							
>350	3	236.2	1.27 (0.41 to 3.94)	1.00			
200 to 350	12	928.5	1.29 (0.73 to 2.28)	0.91 (0.26 to 3.19)	0.88		
<200	44	2832.5	1.55 (1.16 to 2.09)	1.02 (0.32 to 3.26)	0.97		
Peak HIV viral load (copies/mL)							
<100,000	16	1162.1	1.38 (0.84 to 2.25)	1.00			
≥100,000	25	1383.5	1.81 (1.22 to 2.67)	1.22 (0.65 to 2.28)	0.54		
Unknown	18	1451.5	1.24 (0.78 to 1.97)	–			
Current ART adherence^a^							
100%	27	3491.3	0.77 (0.53 to 1.13)	1.00		1.00	
<100%	4	172.2	2.32 (0.87 to 6.19)	3.01 (1.05 to 8.61)	0.04	2.79 (0.92 to 8.45)	0.07
Unknown	28	333.7	8.39 (5.79 to 12.15)	–		–	
Period of treatment switch^a^							
2003 to 2006	13	633.2	2.05 (1.19 to 3.54)	1.00		1.00	
2007 to 2009	11	1131.7	0.97 (0.54 to 1.76)	0.49 (0.22 to 1.08)	0.08	0.87 (0.38 to 1.97)	0.73
2010 to 2012	26	1775.6	1.46 (1.00 to 2.15)	0.61 (0.31 to 1.18)	0.14	1.44 (0.71 to 2.94)	0.31
2013 to 2016	9	456.7	1.97 (1.03 to 3.79)	0.60 (0.25 to 1.46)	0.26	1.41 (0.54 to 3.69)	0.48
Country income status^a^							
High	25	1310.0	1.91 (1.29 to 2.82)	1.00		1.00	
Middle/low	34	2615.0	1.30 (0.93 to 1.82)	0.61 (0.36 to 1.02)	0.06	0.85 (0.46 to 1.59)	0.62

Patient with missing data were included in all analyses, however, HRs for unknown categories are not shown. ART, antiretroviral therapy; VF, virological failure; HR, hazard ratio; NRTI, nucleoside reverse transcriptase inhibitors; NNRTI, non‐nucleoside reverse transcriptase inhibitor; PI, protease inhibitor; INSTI, integrase inhibitors; AE, adverse events.

a Included in final model.

**Table 4:** Crude risk and hazard ratios for virological failure with different patterns of major ART switch

Covariate	VF	Patient years follow up	Rate per 100pt/yrs (95% CI)	Univariate HR (95% CI)	*p*	Multivariate HR (95% CI)	*p*
Type of major ART switch^a^							
NNRTI to PI	12	245.6	4.89 (2.78 to 8.60)	1.00		1.00	
PI to NNRTI	1	284.2	0.35 (0.05 to 2.50)	0.08 (0.01 to 0.61)	0.02	0.13 (0.01 to 1.43)	0.10
PI to INSTI	5	167.4	2.99 (1.24 to 7.18)	0.44 (0.16 to 1.27)	0.13	0.45 (0.11 to 1.75)	0.25
Other	4	74.9	5.34 (2.01 to 14.24)	0.87 (0.28 to 2.68)	0.81	1.05 (0.30 to 3.73)	0.94
AE‐associated switch							
No	9	354.7	2.54 (1.32 to 4.88)	1.00			
Yes	13	417.4	3.11 (1.81 to 5.36)	1.23 (0.52 to 2.88)	0.64		
Baseline age^a^							
Per 5 years older	22	772.0	2.85 (1.88 to 4.33)	1.17 (0.93 to 1.47)	0.19	1.08 (0.85 to 1.38)	0.51
Sex^a^							
Male	20	632.3	3.16 (2.04 to 4.90)	1.00		1.00	
Female	2	139.7	1.43 (0.36 to 5.72)	0.47 (0.11 to 2.09)	0.32	0.42 (0.07 to 2.65)	0.36
HIV exposure							
Heterosexual	8	324.2	2.47 (1.23 to 4.93)	1.00			
Homosexual	12	372.6	3.22 (1.83 to 5.67)	1.19 (0.48 to 2.94)	0.70		
IDU/other	2	75.2	2.66 (0.67 to 10.64)	0.97 (0.20 to 4.64)	0.97		
Hepatitis B surface antigen status							
Negative	16	622.6	2.57 (1.57 to 4.20)	1.00			
Positive	3	55.2	5.43 (1.75 to 16.84)	2.03 (0.63 to 6.56)	0.24		
Unknown	3	94.2	3.18 (1.03 to 9.87)	–			
Hepatitis C antibody status							
Negative	19	652.5	2.91 (1.86 to 4.57)	1.00			
Positive	1	34.9	2.86 (0.40 to 20.33)	1.11 (0.14 to 8.89)	0.92		
Unknown	2	84.7	2.36 (0.59 to 9.45)	–			
Prior AIDS diagnosis^a^							
No	10	497.9	2.01 (1.08 to 3.73)	1.00		1.00	
Yes	12	274.1	4.38 (2.49 to 7.71)	2.30 (1.00 to 5.26)	0.05	2.37 (1.01 to 5.58)	0.05
Current cotrimoxazole use							
No	21	736.0	2.85 (1.86 to 4.38)	1.00			
Yes	1	36.1	2.77 (0.39 to 19.67)	0.71 (0.09 to 5.59)	0.75		
Current CD4 cell count (cells/mm^3^)^a^							
>500	8	433.4	1.85 (0.92 to 3.69)	1.00		1.00	
350 to 500	5	188.9	2.65 (1.10 to 6.36)	1.23 (0.41 to 3.75)	0.71	0.96 (0.29 to 3.20)	0.94
<350	7	124.0	5.64 (2.69 to 11.84)	2.02 (0.74 to 5.49)	0.17	1.01 (0.30 to 3.44)	0.98
Unknown	2	25.8	7.76 (1.94 to 31.02)	–		–	
Nadir CD4 cell count (cells/mm^3^)							
≥200	8	357.0	2.24 (1.12 to 4.48)	1.00			
<200^b^	14	415.0	3.37 (2.00 to 5.70)	1.32 (0.55 to 3.16)	0.53		
Peak HIV viral load (copies/mL)							
<100,000	6	327.8	1.83 (0.82 to 4.07)	1.00			
≥100,000	10	285.2	3.51 (1.89 to 6.52)	1.62 (0.58 to 4.53)	0.36		
Unknown	6	159.1	3.77 (1.69 to 8.39)	–			
Current ART adherence^a^							
100%	12	621.7	1.93 (1.10 to 3.40)	1.00		1.00	
<100%	1	55.9	1.79 (0.25 to 12.69)	0.76 (0.10 to 5.84)	0.79	0.70 (0.09 to 5.63)	0.74
Unknown	9	94.4	9.53 (4.96 to 18.32)	–		–	
Period of treatment switch^a^							
2003 to 2009	6	294.2	2.04 (0.92 to 4.54)	1.00		1.00	
2010 to 2012	13	299.5	4.34 (2.52 to 7.47)	1.42 (0.57 to 3.56)	0.45	1.21 (0.43 to 3.39)	0.72
2013 to 2016	3	178.3	1.68 (0.54 to 5.22)	0.39 (0.10 to 1.51)	0.17	0.43 (0.10 to 1.82)	0.25
Country income status^a^							
High	19	526.7	3.61 (2.30 to 5.66)	1.00		1.00	
Middle/low	3	235.3	1.27 (0.41 to 3.95)	0.40 (0.12 to 1.35)	0.14	0.85 (0.19 to 3.87)	0.83

Patient with missing data were included in all analyses, however, hazard ratios for unknown categories are not shown. ART, antiretroviral therapy; VF, virological failure; HR, hazard ratio; NRTI, nucleoside reverse transcriptase inhibitors; NNRTI, non‐nucleoside reverse transcriptase inhibitor; PI, protease inhibitor; INSTI, integrase inhibitors; AE, adverse events.

a Included in final model.

b Single patient with missing nadir CD4 cell count was included in the <200 cell/mm^3^ category.

## 4 Discussion

The objective of this study was to analyse, in real‐world settings, the rate of VF when substituting ART in virologically suppressed HIV‐infected patients. In our study, the rate of VF during virological suppression was low in patients who had undergone both minor and major ART switches. These results are consistent with prior studies that ART substitution in virologically suppressed HIV‐infected patients is generally effective 20, 21.

Prior studies in clinical trials have proven that switching EFV, for intolerance or toxicity, to NVP or rilpivirine (RPV) is generally safe and efficacious 22, 23. In the open‐label, phase 3b SPIRIT trial concerning 476 patients with no history of VF, switching from a PI‐based regimen to RPV with FTC and TDF proved to be non‐inferior 24. The STRATEGY‐NNRTI trial demonstrated non‐inferiority of ECF/TDF vs. continuation NNRTI with FTC and TDF 13, and the STRATEGY‐PI trial also demonstrated non‐inferiority of simplification to ECF/TDF vs. continuation of ritonavir‐boosted PI with FTC/TCF in virologically suppressed adults 25. In both treatment groups creatinine concentrations increased non‐progressively in patients who switched regimens, as expected because of inhibition of creatinine secretion by cobicistat 26. Treatment discontinuation because of adverse events was rare across both trials 26. The results of our study in non‐clinical trial settings in Asian countries support these findings, with a low frequency of VF and adverse events in patients who experienced minor or major substitution.

Although the rate of VF were generally low in both major and minor substitution groups, patients who had a history of prior AIDS diagnosis in our major substitution analysis had a slightly higher risk of VF compared to those without a history of AIDS (2.37; 95% CI 1.01 to 5.58, *p* = 0.05). In one study with 3447 HIV‐infected patients, protective factors for VF were older age, higher CD4 cell count and medication adherence 27. In another study by Grabar *et al*. considering patients receiving PI‐based therapy, low baseline CD4 cell count and high viral load were both independent predictors of both virological and clinical failure. Neither the type of PI or previous ART taken was associated with risk of clinical progression 28. In our study, neither low nadir CD4 count nor high viral load was associated with increased risk of VF. However, there were only 44 and 14 patients with nadir CD4 cell counts lower than 200 cells/mm^3^ in the minor and major substitution group respectively. The reason for the difference in results may be explained by the smaller number of patients in this study compared to the Grabar *et al*. study, which included 975 patients.

There are several limitations to this study. First, TAHOD participating sites are generally urban referral centres, and each site recruits patients who are considered by local clinicians to have a reasonably good prospect of long‐term follow‐up. This limits the generalizeability of the results, as viral suppression may have been overestimated relative to the background population. Second, the number of patients, especially those patients with major substitutions limited the statistical power of this study. Third, the reasons for changing regimens for each individual were not clearly identifiable from our observational database; thus, we could not draw inferences about whether certain ART switch situations were riskier than others. Finally, patients that did not switch do not have a comparable baseline date as they do not have a date of ART switch. The difference in baseline makes comparison between the three groups difficult.

In a real‐world multisite Asian cohort of cohorts, we found rates of clinical or VF to be low in patients who had undergone both minor and major switches in ART medications. This supports the finding that ART regimen substitution in virologically suppressed patients is effective in non‐clinical trial settings in Asian countries.
